# Beclin-1 Expression Is a Significant Predictor of Survival in Patients with Lymph Node-Positive Gastric Cancer

**DOI:** 10.1371/journal.pone.0045968

**Published:** 2012-09-27

**Authors:** Qi-Rong Geng, Da-Zhi Xu, Long-Jun He, Jia-Bing Lu, Zhi-Wei Zhou, You-Qing Zhan, Yue Lu

**Affiliations:** 1 State Key Laboratory of Oncology in South China, Guangzhou, China; 2 Department of Hematologic Oncology, Sun Yat-sen University Cancer Center, Guangzhou, China; 3 Department of Gastric and Pancreatic Surgery, Sun Yat-sen University Cancer Center, Guangzhou, China; 4 Department of Endoscopy, Sun Yat-sen University Cancer Center, Guangzhou, China; 5 Department of Pathology, Sun Yat-sen University Cancer Center, Guangzhou, China; Pontificia Universidad Catolica de Chile, Chile

## Abstract

**Background:**

Beclin 1 is a main actor of autophagy. The expression of Beclin 1 and its prognostic role in gastric cancer is largely unexplored. The purpose of the present study is to investigate the expression of beclin 1 in gastric cancer cells, tissues and its relationship with prognosis.

**Methods:**

The expression of Beclin 1 was detected in 271 specimens of lymph-node positive gastric cancer patients by immunohistochemistry. The correlation of Beclin 1 expression to clinicopathologic features and survival of gastric cancer was studied. Beclin-1 expression in gastric cancer cell lines and clinical specimens is also detected using reverse transcription-PCR and Western blotting.

**Results:**

Beclin 1 is up-regulated at both mRNA and protein levels in six gastric cancer cell lines compared with those in normal gastric mucosa cell line (GES-1). The expression of Beclin-1 in gastric clinical specimens is also higher than those in the adjacent noncancerous tissues. Of the 271 patients, 229 (84.5%) were Beclin 1 high expression tumors by immunohistochemistry. Beclin 1 expression is closely associated with intravascular embolus. Kaplan-Meier analysis showed high beclin 1 expression was associated with longer overall survival. Both univariate analysis and multivariate analysis revealed that Beclin 1 expression were independent prognostic factors in the patients with node-positive gastric cancer.

**Conclusions:**

Our findings strongly suggest that Beclin 1 has a potential role in tumorigenesis of gastric cancer and could be a promising biomarker for predicting the prognosis of patients with lymph node-positive gastric cancer. It might also serve as a novel therapeutic target for gastric cancer treatment.

## Introduction

Gastric cancer remains a major public health problem worldwide, specially in East Asian countries, the age-standardized incidence rate is >20 per 100,000 [1]. For the advanced gastric cancer, its prognosis is still poor, with an estimated overall 5-year survival rate of 25% or less [2], [3]. Lymph node metastasis is the most powerful prognostic indicator following curative resection [4]–[7]. About 75% of advanced patients have node-positive gastric cancer, whose prognosis were significantly worse than for those with node-negative disease [4].

In previous studies, we have found positive lymph node ratio is an independent prognostic indicator to the patients with node-positive gastric cancer and intraperitoneal chemotherapy may be beneficial [8], [9]. However, a fundamental step toward improving the survival of patients with lymph node-positive gastric cancer lies in the increased understanding of the tumor biological behavior and searching for the possible targets for individual therapy [10], [11]. For example, the human epidermal growth factor receptor 2 (HER2) has become now a new marker of gastric cancer following evidence-based principles [12].Adding trastuzumab to standard chemotherapy could change the poor survival of patients with HER2-positive metastatic gastric cancer [13]–[15].

Recently, the role of autophagy in cancer development and cancer treatment has been given great concern [16]–[18]. Beclin 1, a key regulator of autophagy formation, was found overexpression in a variety of human cancers [19]–[22].

However, there are still relatively few studies that have investigated the association between Beclin 1 and gastric cancer. In particular, no data regarding their effects on prognosis in gastric cancer, have yet been reported.

The present study investigated Beclin 1 expression in gastric cancer cells, tissues and its clinicopathologic significance in patients with lymph node-positive gastric cancer. Furthermore, we analyzed the relationships between the Beclin 1 expression with the prognosis to determine whether Beclin 1 can predict clinical outcome.

## Materials and Methods

### Cell lines and culture conditions

Human gastric cancer cell lines HGC-27, MKN803,MGC-803,SGC-7901,MKN-28,BGC-823 were gifts from Peking University School of Oncology (Beijing, P.R. China.) [23], [24]. The normal gastric mucosa cell line, GES-1, derived from a human fetal gastric mucosa epithelium, was obtained from the First Affiliated Hospital Sun Yat-Sen University [25]. These cell lines were maintained in RPMI 1640 medium (Invitrogen) supplemented with 10% fetal bovine serum (Hyclone), penicillin (100 units/mL) and streptomycin (100 units/mL) at 37°C and 5% CO2 in a humidified incubator.

### Reverse transcription-PCR

Total RNA was extracted by using TRIzol method. The RNA was pretreated with DNase and used for cDNA synthesis with random hexamers. The mixture (25 µL total) for PCR consisted of 0.5 µl cDNA, 0.5 U Taq DNA polymerase, 2.5 µl of 10× PCR buffer, 2.5 mM dNTP mixture, and 50 pM sense and antisense primers each. Beclin 1 were analyzed by following primers: Beclin 1 5′CGTGGAATGGAATGAGAT3′

reverse primer 5′ GTAAGGAACAAGTCGGTAT3′;

Actin: 5′AGCCATGTACGTAGCCATCC3′

and 5′ GTGGTGGTGAAGCTGTAGC 3′.

### Western blot analysis

Cells were lysed in lysis buffer and the concentration of protein was determined by the Bradford dye method (Bio-Rad Laboratories). Equal amounts of cell extract were subjected to SDS-PAGE and transferred to PVDF membrane (Bio-Rad). Expression of Beclin 1 was determined with a rabbit monoclonal antibody (1∶1000, Novus Biologicals, NB500-249) according to the manufacturer's suggested protocols.

### Patients studied

The study consisted of 271 gastric cancer patients who underwent radical resection for histologically confirmed gastric carcinoma from Cancer Center of Sun Yat-sen University between January 1998 and December 2006. We obtained informed consent from all participants involved in this study. Ethical approval was obtained from Sun Yat-sen University Cancer Center research ethics committee. The eligibility criteria included histologically confirmed R0 resection, which was defined as no macroscopic and microscopic residual tumor and postoperative survival time ≥6 months. Patients with distant metastases or preoperative therapy or lymph node-negative were excluded from the study. The 7thTNM classification by the American Joint Committee on Cancer and International Union Against Cancer criteria was used for pathologic staging [26].

All patients had follow-up after surgery at 6 to 12 month intervals; Median follow up period was 38 (range 10 to 145) months for all patients. Overall survival was defined as was defined as the period between the time of surgery and death or was censored at the last follow-up.

### Immunohistochemistry

Beclin 1 was detected with a rabbit monoclonal antibody (1∶100, Novus Biologicals, NB500-249). All tumor specimens were obtained from surgically resected gastric cancers before adjuvant therapy. Formalin-fixed, paraffin-embedded tissue blocks were stored at room temperature identified by an identification number. The procedures were done similarly to the method previously described [23]. The immunostained sections were evaluated and tissues from human prostate adenocarcinomas served as positive control. All slides were evaluated by two independent observers based on the proportion of positively stained tumor cells and intensity of staining. According to recently described criteria for rating Beclin 1 expression, the intensity of membrane staining varied from weak to strong: 0(no staining), 1(weak staining), 2 (moderate staining) and and 3 (intense staining) [27]. The percentage of positive tumor cells was scored as 0 (<5%), 1 (6% to 25%), 2 (26% to 50%), 3 (51% to 70%) and 4 (>70%). Staining index was calculated by totaling the staining intensity score and the percentage of positive cells score. Immunoreactive score equal or higher than 4 was used to classify tumors with high Beclin 1 expression, and a staining score below 4 was used to indicate low Beclin 1 expression.

### Statistical analyses

All statistical analyses were carried out using SPSS software (version 18 for Windows; SPSS, Chicago, IL). Differences at P<.05 were considered to be statistically significant. The association of Beclin-1 expression with various clinicalopathologic features was analyzed using the chi-square test. Survival curves were plotted by the Kaplan-Meier method and compared by the log-rank test. A Cox proportional hazard model (backward, stepwise) for multivariable analysis was applied for factors that achieved significance in univariable analysis.

## Results

### Beclin 1 expression in gastric cancer cell lines

**Figure 1:**
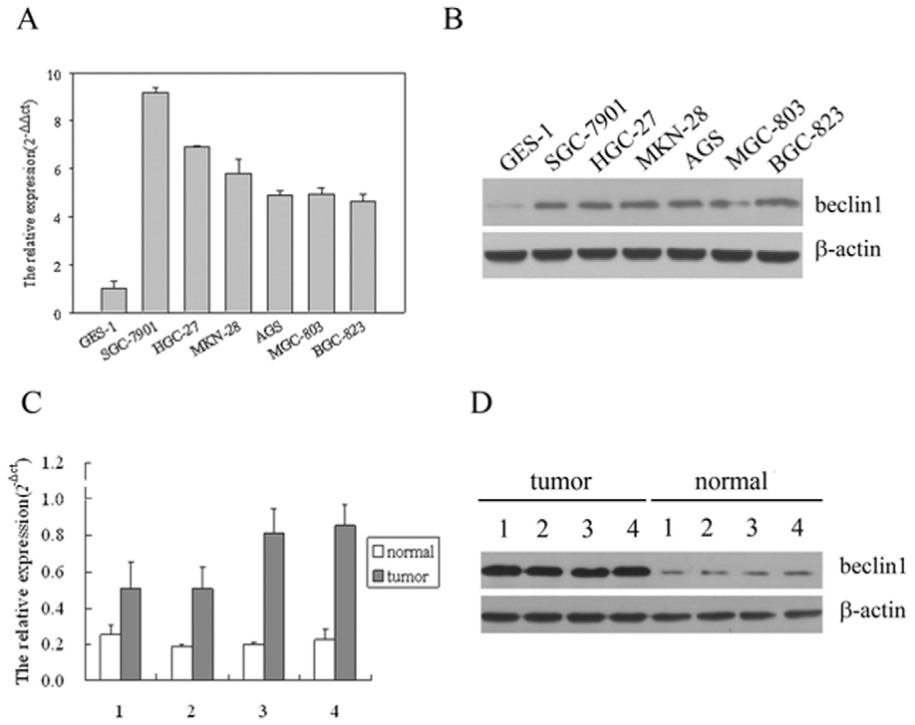
Expression of Beclin 1 mRNA and protein in cells and tissues. (A) Beclin 1 mRNA expression level up-regulated in gastric cancer cell lines compared with normal gastric epithelial cells by reverse transcription-PCR. (B) Beclin 1 protein expression level up-regulated in gastric cancer cell lines compared with normal gastric epithelial cells by western blotting. (C) Beclin 1 mRNA expression is elevated in primary gastric tumors compared with paired gastric adjacent noncancerous tissues by reverse transcription-PCR. (D) Beclin 1 protein expression is elevated in primary gastric tumors compared with paired gastric adjacent noncancerous tissues by western blotting.

Overexpression of Beclin 1 has been reported in many cancers. Its expression status in gastric cancer cell lines, however, remains unclear. To determine the expression levels of beclin-1 transcripts and protein, semi-quantitative reverse transcription-PCR analysis and western blotting analysis were conducted separately in normal human gastric epithelial cells (GES-1) and six gastric cancer cell lines (HGC-27,MKN803,MGC-803,SGC-7901, MKN-28,BGC-823). We found that Beclin 1 mRNA and protein expression level up-regulated in all six gastric cancer cell lines compared with normal gastric epithelial cells GES-1. (Fig. 1 A and B)

### Beclin 1 expression in gastric cancer tissues

To determine beclin-1 mRNA and protein expression in gastric cancer tissues, 4 paired gastric cancer tissues and noncancerous tissues adjacent to cancer lesions were detected by reverse transcription-PCR analysis and western blotting analysis, separately. It was found that Beclin-1 in all four tumor tissue specimens had a significantly higher expression at both mRNA and protein levels than those in the normal tissue adjacent to tumors from the same patients (Fig. 1 C and D).

### Beclin 1 expression in gastric cancer

**Figure 2:**
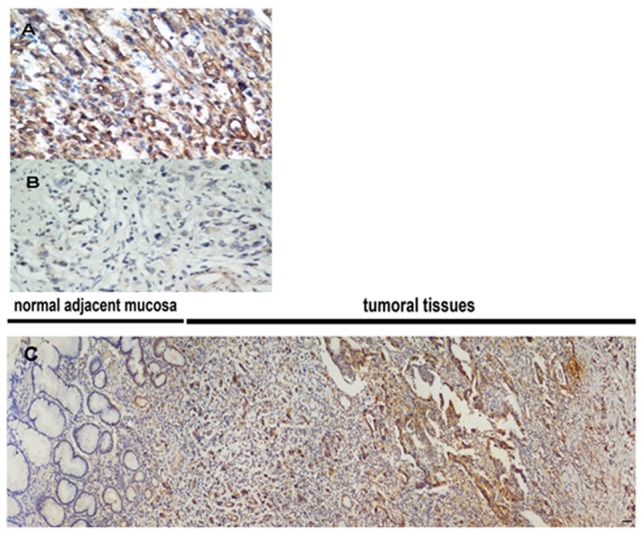
Representative examples of Beclin1 expression. High Beclin 1 (A) and low Beclin 1 (B) expression in human gastric cancer specimens (×400), Beclin 1 positive staining in the tumor and negative staining in normal adjacent mucosa(×20) (C).

To investigate whether beclin-1 could clinically correlate with gastric cancer progression, expression and subcellular localization of Beclin 1 protein was detected by immunohistochemistry in 271 paraffin-embedded gastric cancer tissues. Specific Beclin 1 staining was mostly found on the membrane-plasma and cytoplasm in the cancer cells, occasionally the nucleus. Figure 2 shows representative examples of Beclin 1 immunostaining. Of the 271 patients, 229 (84.5%) were Beclin 1 high expression tumors.

### Survival analysis

**Figure 3:**
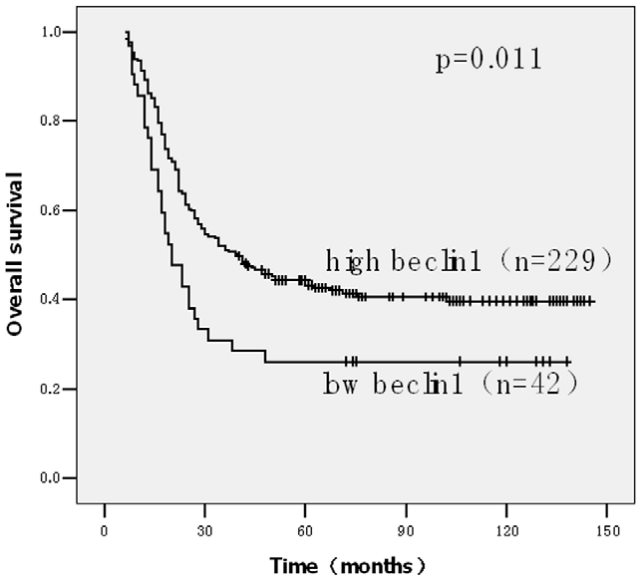
Kaplan-Meier estimates of the probability of survival. Patients with high expression Beclin 1 showed significantly longer overall survival than those with Beclin 1 low expression. (p = .011).

**Figure 4:**
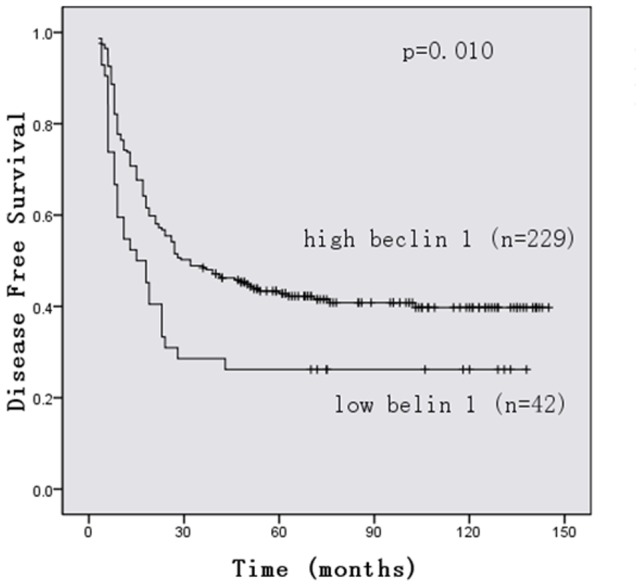
Kaplan-Meier estimates of the probability of disease free survival (DFS). Patients with high expression Beclin 1 showed significantly longer DFS than those with Beclin 1 low expression. (p = .010).

Patients with Beclin 1 low expression gastric cancer showed significantly shorter 5-year overall survival rates (OS, p = .011; Fig. 3) and disease free survival (DFS, p = .010; Fig. 4) than those with Beclin 1 high expression ones. The expression of Beclin 1 is closely associated with intravascular embolus (P =  0.017). However, there was no significant correlation between the expression level of Beclin 1 and age, sex, site, tumor size, T classification, N classification, or pathologic stage of gastric cancer patients (Table 1). Beclin 1 expression, site, intravascular embolus, pathologic T classification, pathologic N classification and pathologic stage were independent prognostic factors in univariate analysis (data not shown).In multivariable analysis, Beclin 1 expression, intravascular embolus, site and pathologic stage were significant independent prognostic factor for survival time (Table 2).

**Table 1:** Clinicopathologic correlation of beclin1 expression in gastric cancer.

Factors	No. of patients (%)		*p*
	Beclin 1 low expression	Beclin 1 high expression	
Age (y)			0.402
<60	19	122	
≥60	23	107	
Sex			0.465
Male	28	165	
Female	14	64	
Site			0.343
**Cardia-fundus**	20	132	
**Corpus**	8	33	
**Antrum**	11	58	
**two or more sites**	3	6	
Tumor size			0.401
≤4 cm	11	75	
>4 cm	32	153	
Intravascular embolus			0.017
No	34	212	
Yes	8	17	
Pathologic T classification			0.917
T2	3	18	
T3	8	52	
T4a	25	133	
T4b	6	26	
Pathologic N classification			0.620
N1	12	76	
N2	14	89	
N3a	12	47	
N3b	4	17	
Pathologic stage (pTNM)			0.441
IIA	2	6	
IIB	4	31	
IIIA	7	62	
IIIB	14	70	
IIIC	15	60	

**Table 2:** Univariate and multivariate analysis of different prognostic variables in patients with gastric cancer by cox regression analysis.

	Univariate analysis			Multivariate analysis	
	No. patients	*P*	Regression coefficient (SE)	*P*	Relative risk (95% confidence interval)
Beclin1		0.013		0.044	
negative	42		−0.495(0.200)		0.663(0.444–0.989)
positive	229				
Site		0.000		0.002	
upper	152				
Middle	41	0.542	0.128(0.210)	0.711	0.923(0.603–1.412)
lower	69	0.032	−0.436(0.203)	0.007	0.574(0.384–0.859)
Diffuse	9	0.001	1.213(0.354)	0.025	2.275(1.106–4.680)
Intravascular embolus		0.000		0.000	
No	246		0.996(0.232)		2.862(1.776–4.613)
Yes	25				
Pathologic stage (pTNM)		0.000		0.000	
IIA	8				
IIB	35	0.529	0.396(0.629)	0.313	1.890(0.549–6.509)
IIIA	69	0.406	0.501(0.603)	0.168	2.313(0.703–7.615)
IIIB	84	0.118	0.928(0.594)	0.059	3.079(0.957–9.904)
IIIC	75	0.018	1.406(0.593)	0.005	5.295(1.637–17.124)

## Discussion

Autophagy is a self-degradation mechanism and associated with tumor progression. Beclin 1, a mammalian orthologue of yeast Atg6, plays a crucial role in the vesicle nucleation process of autophagy [28]. It is a gene indispensable for the first phases of autophagy [29]–[30].

The current study has, for the first time, showed Beclin 1 is up-regulated at both mRNA and protein levels in six gastric cancer cell lines compared with those in normal gastric epithelial cells GES-1. We also revealed Beclin 1 expression is elevated in primary gastric tumors compared with four paired gastric cancer samples adjacent to tumors from the same patients by reverse transcription-PCR and western blotting. The results differ from those reported in liver cancer cells and cervical cancer cells, which showed Beclin 1 expression significantly lower compared to the corresponding normal tissues [31], [32]. The possible explaination is that beclin-1 expression in gastric cancers contribute to the induction of autophagic cell death, thereby producing selective pressure to override cell death during development the cancer [27], [33].

In present study, we observed that 229 (84.5%) patients had high expression Beclin 1 in 271 gastric cancer patients, in line with the finding of the previous study by Ahn et al.Their data showed no or very weak expression of Beclin1 in normal mucosal cells of stomach by immunohistochemistry [27]. However, they did not explore the beclin-1 expression at mRNA level in cell lines, nor investigate its role in the prognosis.

We then analyzed the relationship between the expression of beclin-1 and clinical characteristics of the patients. There was no significant correlation between the expression of beclin-1 and age, gender, T classification, N classification, or stage of patients with gastric cancer. Interesting, in our current study, there was a significant relationship between the expression of beclin-1 and intravascular embolus. It suggesting that beclin-1 could be used as a marker intravascular embolus in the patients with node-positive gastric cancer. These observations highlight the important role of beclin-1 in the development of gastric cancer.

Furthermore, we found low beclin-1 expression was strongly associated with decreased survival in gastric cancer patients (*P* = 0.011) and the intravascular embolus subset (*P* = 0.017). In multivariable analysis, Beclin-1 expression retained its independence as a prognostic factor(*P* = 0.044). These results suggest a potentially promising usefulness of beclin-1 as a prognostic and survival indicator. The mechanism is still not clear. The alternative explanation for this may be that beclin-1 expression in gastric cancers limits chromosomal instability and decreases the frequency of additional mutation [34].

To our knowledge, the study is the first report that Beclin 1 had a significantly higher expression in gastric cancer cells and tissues at both mRNA and protein levels compared to their normal counterparts. Moreover, we found high beclin 1 expression was associated with longer overall survival. Both univariate analysis and multivariate analysis showed Beclin 1 expression, site, intravascular embolus and pathologic stage were independent prognostic factors. The results strongly suggest that Beclin 1 has a potential role in tumorigenesis of gastric cancer. It could be a promising biomarker and a novel therapeutic strategies to patients with lymph node-positive gastric cancer. Further studies are also needed to clarify the mechanism by which autophagy actor Beclin 1 is involved in the development and progression of gastric cancer.
